# Tetra­kis(μ-4-chloro­benzoato-κ^2^ *O*:*O*′)bis­[(ethanol-κ*O*)copper(II)](*Cu*—*Cu*)

**DOI:** 10.1107/S1600536813006909

**Published:** 2013-03-16

**Authors:** Viviana Mollica Nardo, Francesco Nicoló, Alessandro Saccà, Giuseppe Bruno, Ileana Ielo

**Affiliations:** a Dipartimento di Scienze Chimiche, Universitá di Messina, Viale F. Stagno d’Alcontres 31, 98166 Messina, Italy

## Abstract

In the centrosymmetric dinuclear title Cu^II^ complex, [Cu_2_(C_7_H_4_ClO_2_)(C_2_H_5_OH)_2_], the Cu—Cu distance is 2.5905 (4) Å. The two metal atoms are bridged by four 4-chloro­benzoate ligands and each has an ethanol mol­ecule in the axial position of the overall octahedral coordination environment. The crystal packing features O—H⋯O hydrogen bonds.

## Related literature

For general background to metal-coordination polymers, see: Deka *et al.* (2006 ▶); Eddaoudi *et al.* (2001 ▶); Casarin *et al.* (2005 ▶). For their coordination modes, see: Chen & Chen (2002 ▶). For related structures, see: Hauptmann *et al.* (2000 ▶); Ueda *et al.* (2005 ▶); Hökelek *et al.* (2008 ▶); Hu *et al.* (2004 ▶).
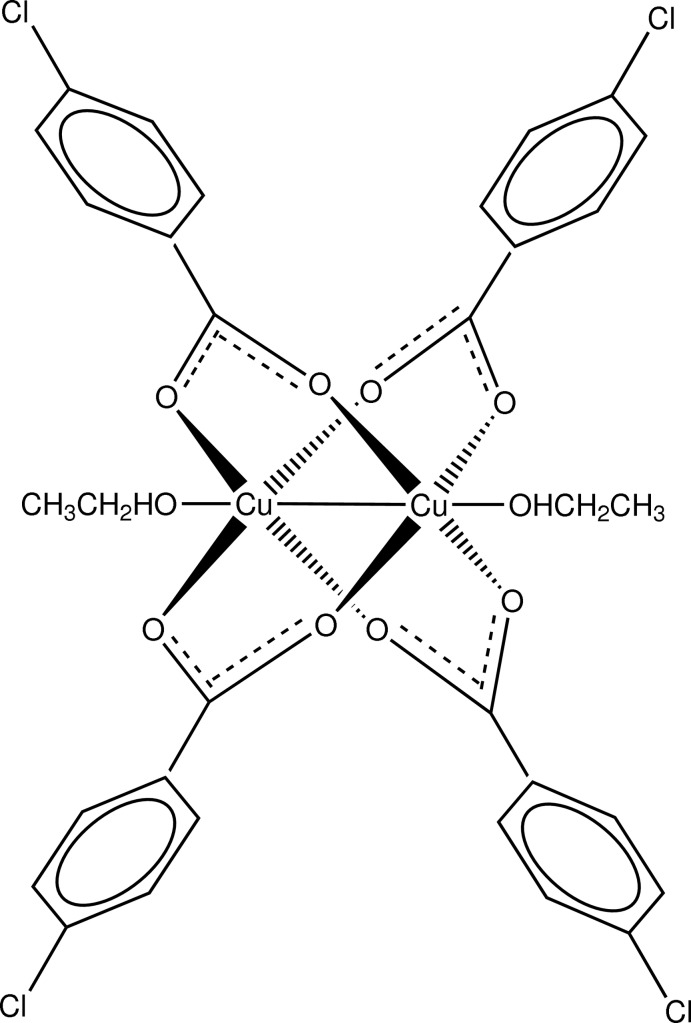


## Experimental

### 

#### Crystal data

[Cu_2_(C_7_H_4_ClO_2_)(C_2_H_6_O)_2_]
*M* *_r_* = 841.42
Triclinic, 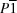

*a* = 6.5766 (1) Å
*b* = 11.1792 (2) Å
*c* = 12.4355 (3) Å
α = 93.175 (1)°
β = 103.890 (1)°
γ = 98.688 (1)°
*V* = 873.34 (3) Å^3^
*Z* = 1
Mo *K*α radiation
μ = 1.58 mm^−1^
*T* = 296 K
0.38 × 0.18 × 0.10 mm

#### Data collection

Bruker APEXII CCD diffractometer
Absorption correction: multi-scan (*SADABS*; Bruker, 2007 ▶) *T*_min_ = 0.695, *T*_max_ = 0.746
36853 measured reflections
5070 independent reflections
4288 reflections with *I* > 2σ(*I*)
*R*_int_ = 0.033

#### Refinement

*R*[*F* ^2^ > 2σ(*F* ^2^)] = 0.037
*wR*(*F* ^2^) = 0.102
*S* = 1.03
5070 reflections
220 parameters
H-atom parameters constrained
Δρ_max_ = 0.50 e Å^−3^
Δρ_min_ = −0.42 e Å^−3^

### 

Data collection: *APEX2* (Bruker, 2007 ▶); cell refinement: *SAINT* (Bruker, 2007 ▶); data reduction: *SAINT*; program(s) used to solve structure: *SHELXS97* (Sheldrick, 2008 ▶); program(s) used to refine structure: *SHELXL97* (Sheldrick, 2008 ▶); molecular graphics: *XPW* (Siemens, 1996 ▶); software used to prepare material for publication: *SHELXTL* (Sheldrick, 2008 ▶).

## Supplementary Material

Crystal structure: contains datablock(s) global, I. DOI: 10.1107/S1600536813006909/fj2617sup1.cif

Structure factors: contains datablock(s) I. DOI: 10.1107/S1600536813006909/fj2617Isup2.hkl

Additional supplementary materials:  crystallographic information; 3D view; checkCIF report

## Figures and Tables

**Table 1:** Hydrogen-bond geometry (Å, °)

*D*—H⋯*A*	*D*—H	H⋯*A*	*D*⋯*A*	*D*—H⋯*A*
O5—H5⋯O2^i^	0.82	2.35	3.073 (3)	148
O5—H5⋯O3^ii^	0.82	2.57	3.047 (3)	118

Symmetry codes: (i) 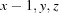; (ii) 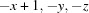.

**Fig. 1.**
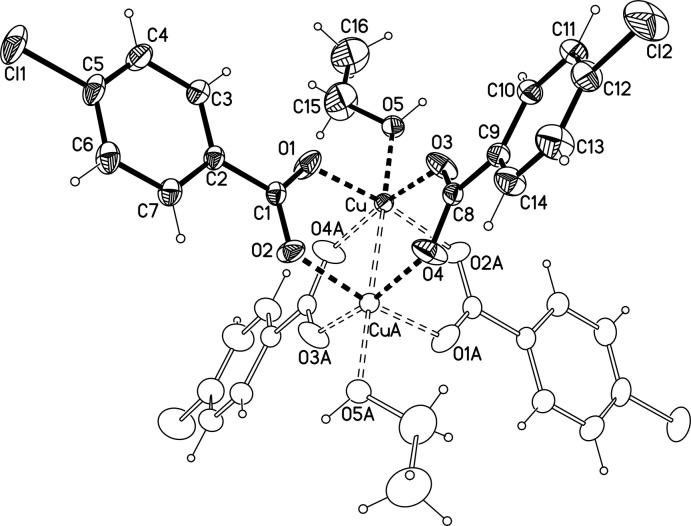
Perspective view of the title molecule with the corresponding atom labelling of the crystallographic asymmetric unit. The equivalent centrosymmetric fragment (2 - x, -y, -z) is represented by empty style. Displacement ellipsoids are plotted at a 30% probability level while H atom size is arbitrary.

**Fig. 2.**
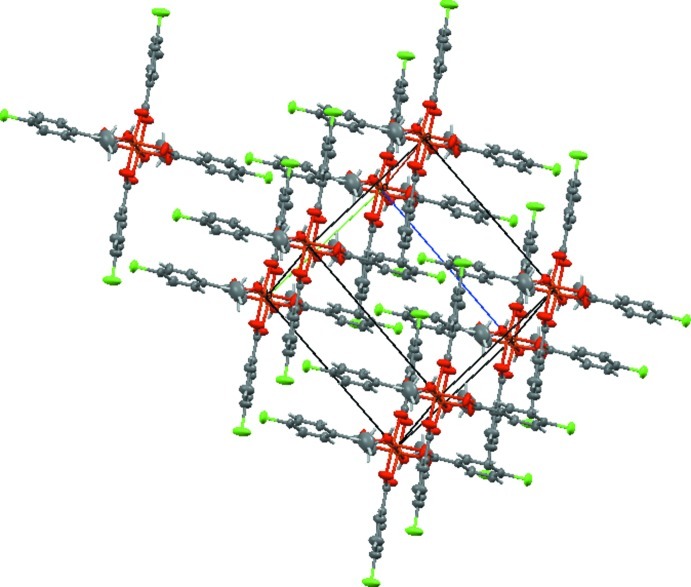
Crystal packing of the dinuclear units showing the centrosymmetric arrangement of each complex couple (3 - x, -y, 1 - z) assembled by weak π-stacking and chlorine-phenyl interactions. Displacement ellipsoids are plotted at a 30% probability level and H atoms are drawn with an arbitrary radius.

## supplementary crystallographic information

### Comment

The chemistry of metal-coordination polymers has been advanced due to their diverse topologies and potential applications in smart optoelectronic, magnetic, microporous and biomimetic materials with specific structures, properties, and reactivities (Deka *et al.*, 2006; Eddaoudi *et al.*, 2001). A coordination polymer contains one or more center of metal linked by coordinated ligands into an infinite array, in one/two or three dimension. Coordination polymers constitute one of the most important classes of organic–inorganic hybrid materials (Casarin *et al.*, 2005) that have been the subject of intensive research in recent years. In this paper is presented a new dinuclear complex of copper(II).

The carboxylates ligands have different coordination modes so it is important to have control on the binding of carboxylate to a metal ion in specific manner in the presence of other ligand/s. Some benzoic acid derivatives, such as 4-aminobenzoic acid, have been extensively reported in coordination chemistry, as bifunctional organic ligands, due to the varieties of their coordination modes (Chen & Chen, 2002; Hauptmann *et al.*, 2000). In this compound the copper have a octahedral coordination and combine another center of copper, four oxygen of four acid and one ethanol. The copper dimer has a perfect C*i* symmetry and is placed on a crystallographic centre of inversion (Figure 1). The mean value of the distances Cu—O [1.953 (2) Å] is similar to that reported in other structures (Ueda *et al.*, 2005; Hökelek *et al.*, 2008). The structure is very similar to another already known (Hu *et al.*, 2004) differing for the halogen atom bonded to the carboxylic acid.

The crystal packing is constituted by centrosymmetric couple (3 - *x*, -*y*, 1 - *z*) of the dinuclear complexes assembled by weak π-stacking interaction of the C2—C7 aromatic rings [C5 at 3.586 (4) Å from centroid of the other ring] and a chlorine Cl1 interaction with the C9—C14 ring of the other molecule [Cl1—C9 is 3.387 (3) Å] (Figure 2). The packing is further stabilized by intermolecular hydrogen bonds (Tables 1).

### Experimental

The reagents used here were purchased from commercial sources (Sigma–Aldrich). To synthetize the title complex 1 ml of Copper(II) Chloride (0,1 mmol; 100mM) were added to a water (3 ml) / ethanol (3 ml) mixture containing 0,0134 g of melanine and 0,017 g of acid *p*-Chlorobenzoic. The reaction was sealed in a Teflon-lined stainless steel autoclave, which was heated at 130°C for 3 days under autogenous pressure. After slow cooling to room temperature in 6 h. In this conditions are formed green crystals of the complex and colourless crystal of melamine. A suitable sample of the green crystals has been selected by optical microscope and used in X-Ray structure determination.

### Refinement

The structure was solved by direct method and subsequent Fourier difference techniques and refined using full-matrix least-squares procedure on F^2^ with anisotropic thermal parameters for all non-hydrogen atoms (*SHELXL97*). All non hydrogen atoms were refined anisotropically. Several hydrogen atoms were located on the final difference map, the H atoms were included in the refinement *via* the "riding model" method with the *X*—H bond geometry and the H isotropic displacement parameter depending on the parent atom *X*. Owing to the usual conformational disorder of the terminal ethanol, no suitable bond/angles constrain was introduced during the last refinement cycles so that the ligand geometry appears distorted but similar to the reported values in analogous complexes.

### Crystal data

**Table:**

[Cu_2_(C_7_H_4_ClO_2_)(C_2_H_6_O)_2_]	*Z* = 1
*M**_r_* = 841.42	*F*(000) = 426
Triclinic, *P*1	*D*_x_ = 1.600 Mg m^−^^3^
Hall symbol: -P 1	Mo *K*α radiation, λ = 0.71073 Å
*a* = 6.5766 (1) Å	Cell parameters from 9962 reflections
*b* = 11.1792 (2) Å	θ = 2.6–28.9°
*c* = 12.4355 (3) Å	µ = 1.58 mm^−^^1^
α = 93.175 (1)°	*T* = 296 K
β = 103.890 (1)°	Irregular, green
γ = 98.688 (1)°	0.38 × 0.18 × 0.10 mm
*V* = 873.34 (3) Å^3^	

### Data collection

**Table:**

Bruker APEXII CCD diffractometer	5070 independent reflections
Radiation source: fine-focus sealed tube	4288 reflections with *I* > 2σ(*I*)
Graphite monochromator	*R*_int_ = 0.033
φ and ω scans	θ_max_ = 30.1°, θ_min_ = 3.2°
Absorption correction: multi-scan (*SADABS*; Bruker, 2007)	*h* = −9→9
*T*_min_ = 0.695, *T*_max_ = 0.746	*k* = −15→15
36853 measured reflections	*l* = −17→17

### Refinement

**Table:**

Refinement on *F*^2^	Primary atom site location: structure-invariant direct methods
Least-squares matrix: full	Secondary atom site location: difference Fourier map
*R*[*F*^2^ > 2σ(*F*^2^)] = 0.037	Hydrogen site location: inferred from neighbouring sites
*wR*(*F*^2^) = 0.102	H-atom parameters constrained
*S* = 1.03	*w* = 1/[σ^2^(*F*_o_^2^) + (0.0523*P*)^2^ + 0.4776*P*] where *P* = (*F*_o_^2^ + 2*F*_c_^2^)/3
5070 reflections	(Δ/σ)_max_ = 0.004
220 parameters	Δρ_max_ = 0.50 e Å^−^^3^
0 restraints	Δρ_min_ = −0.42 e Å^−^^3^

### Special details

**Table:**

Experimental. SADABS-2008/1 - Bruker AXS area detector scaling and absorption correction. wR2(int) was 0.0889 before and 0.0361 after correction.The crystals suitable for the X-ray analysis were obtained by solvothermal synthesis of the reaction of *p*-Chlorobenzoate with Copper(II) salt in water/ethanol (1:1) mixed together with melanin.
Geometry. All e.s.d.'s (except the e.s.d. in the dihedral angle between two l.s. planes) are estimated using the full covariance matrix. The cell e.s.d.'s are taken into account individually in the estimation of e.s.d.'s in distances, angles and torsion angles; correlations between e.s.d.'s in cell parameters are only used when they are defined by crystal symmetry. An approximate (isotropic) treatment of cell e.s.d.'s is used for estimating e.s.d.'s involving l.s. planes.
Refinement. Refinement of *F*^2^ against ALL reflections. The weighted *R*-factor *wR* and goodness of fit *S* are based on *F*^2^. Conventional *R*-factors *R* are based on *F*, with *F* set to zero for negative *F*^2^. The threshold expression of *F*^2^ > 2σ(*F*^2^) is used only for calculating *R*-factors(gt) *etc*. and is not relevant to the choice of reflections for refinement. *R*-factors based on *F*^2^ are statistically about twice as large as those based on *F*, and *R*- factors based on ALL data will be even larger.

### Fractional atomic coordinates and isotropic or equivalent isotropic displacement parameters (Å^2^)

**Table:**

	*x*	*y*	*z*	*U*_iso_*/*U*_eq_	
Cu1	0.83458 (4)	−0.06609 (2)	0.018444 (19)	0.02666 (8)	
O1	1.0314 (3)	−0.10047 (19)	0.15327 (16)	0.0579 (5)	
O2	1.3138 (3)	0.00860 (19)	0.11950 (14)	0.0505 (5)	
C1	1.2246 (3)	−0.05778 (18)	0.17776 (17)	0.0314 (4)	
C2	1.3598 (3)	−0.08960 (19)	0.28342 (17)	0.0327 (4)	
C3	1.2696 (4)	−0.1556 (2)	0.3558 (2)	0.0430 (5)	
H3	1.1231	−0.1805	0.3386	0.052*	
C4	1.3950 (5)	−0.1850 (3)	0.4537 (2)	0.0519 (6)	
H4	1.3341	−0.2293	0.5026	0.062*	
C5	1.6119 (5)	−0.1476 (2)	0.47747 (19)	0.0473 (6)	
C6	1.7056 (4)	−0.0834 (2)	0.4065 (2)	0.0470 (6)	
H6	1.8524	−0.0603	0.4236	0.056*	
C7	1.5796 (4)	−0.0530 (2)	0.30873 (19)	0.0404 (5)	
H7	1.6414	−0.0085	0.2604	0.048*	
Cl1	1.77151 (15)	−0.18454 (8)	0.59985 (6)	0.0714 (2)	
O3	0.8063 (3)	0.08393 (16)	0.09854 (16)	0.0508 (5)	
O4	1.0913 (3)	0.19567 (16)	0.06969 (19)	0.0601 (6)	
C8	0.9365 (3)	0.18035 (19)	0.11129 (16)	0.0318 (4)	
C9	0.9026 (4)	0.28429 (19)	0.18188 (18)	0.0348 (4)	
C10	0.7242 (4)	0.2754 (2)	0.2238 (2)	0.0407 (5)	
H10	0.6230	0.2049	0.2069	0.049*	
C11	0.6966 (5)	0.3717 (2)	0.2911 (2)	0.0503 (6)	
H11	0.5771	0.3663	0.3193	0.060*	
C12	0.8467 (6)	0.4743 (2)	0.3154 (2)	0.0579 (7)	
C13	1.0244 (6)	0.4853 (3)	0.2747 (3)	0.0663 (8)	
H13	1.1250	0.5561	0.2922	0.080*	
C14	1.0514 (5)	0.3897 (2)	0.2074 (2)	0.0512 (6)	
H14	1.1707	0.3963	0.1790	0.061*	
Cl2	0.8148 (2)	0.59368 (9)	0.40221 (10)	0.0981 (4)	
O5	0.5654 (2)	−0.17863 (14)	0.04837 (14)	0.0397 (3)	
H5	0.4613	−0.1549	0.0620	0.060*	
C15	0.5781 (8)	−0.2948 (4)	0.0851 (5)	0.1087 (17)	
H15A	0.6283	−0.3412	0.0316	0.130*	
H15B	0.6867	−0.2846	0.1548	0.130*	
C16	0.3962 (8)	−0.3657 (4)	0.1019 (5)	0.1163 (18)	
H16A	0.4349	−0.4090	0.1663	0.174*	
H16B	0.3314	−0.4227	0.0378	0.174*	
H16C	0.2975	−0.3141	0.1132	0.174*	

### Atomic displacement parameters (Å^2^)

**Table:**

	*U*^11^	*U*^22^	*U*^33^	*U*^12^	*U*^13^	*U*^23^
Cu1	0.02393 (12)	0.02850 (13)	0.02705 (13)	0.00328 (8)	0.00589 (8)	0.00402 (8)
O1	0.0326 (8)	0.0750 (13)	0.0550 (11)	−0.0070 (8)	−0.0070 (7)	0.0366 (10)
O2	0.0298 (8)	0.0832 (13)	0.0354 (8)	0.0014 (8)	0.0030 (6)	0.0257 (9)
C1	0.0303 (9)	0.0317 (9)	0.0305 (10)	0.0082 (8)	0.0029 (8)	0.0016 (8)
C2	0.0348 (10)	0.0335 (10)	0.0280 (9)	0.0098 (8)	0.0025 (8)	0.0013 (8)
C3	0.0413 (12)	0.0516 (13)	0.0373 (12)	0.0128 (10)	0.0077 (9)	0.0110 (10)
C4	0.0640 (17)	0.0570 (15)	0.0372 (13)	0.0175 (13)	0.0101 (11)	0.0165 (11)
C5	0.0621 (16)	0.0478 (13)	0.0279 (11)	0.0255 (12)	−0.0055 (10)	0.0002 (9)
C6	0.0396 (12)	0.0535 (14)	0.0394 (12)	0.0118 (10)	−0.0073 (10)	−0.0024 (10)
C7	0.0356 (11)	0.0462 (12)	0.0349 (11)	0.0072 (9)	0.0001 (9)	0.0034 (9)
Cl1	0.0926 (6)	0.0776 (5)	0.0369 (3)	0.0401 (4)	−0.0136 (3)	0.0065 (3)
O3	0.0396 (9)	0.0434 (9)	0.0689 (12)	−0.0044 (7)	0.0257 (8)	−0.0206 (8)
O4	0.0695 (12)	0.0341 (8)	0.0897 (15)	−0.0028 (8)	0.0556 (12)	−0.0083 (9)
C8	0.0324 (10)	0.0350 (10)	0.0287 (9)	0.0106 (8)	0.0056 (8)	0.0046 (8)
C9	0.0398 (11)	0.0344 (10)	0.0310 (10)	0.0099 (8)	0.0084 (8)	0.0024 (8)
C10	0.0451 (12)	0.0394 (11)	0.0407 (12)	0.0100 (9)	0.0152 (10)	0.0024 (9)
C11	0.0614 (16)	0.0510 (14)	0.0484 (14)	0.0202 (12)	0.0264 (12)	0.0032 (11)
C12	0.086 (2)	0.0420 (13)	0.0532 (16)	0.0204 (14)	0.0275 (15)	−0.0057 (12)
C13	0.080 (2)	0.0396 (14)	0.079 (2)	−0.0028 (14)	0.0324 (18)	−0.0152 (14)
C14	0.0545 (15)	0.0406 (13)	0.0612 (16)	0.0033 (11)	0.0251 (13)	−0.0039 (11)
Cl2	0.1417 (10)	0.0610 (5)	0.1054 (8)	0.0210 (6)	0.0633 (7)	−0.0266 (5)
O5	0.0338 (8)	0.0368 (8)	0.0509 (9)	0.0031 (6)	0.0159 (7)	0.0103 (7)
C15	0.102 (3)	0.060 (2)	0.181 (5)	0.008 (2)	0.061 (3)	0.056 (3)
C16	0.114 (4)	0.081 (3)	0.161 (5)	−0.006 (3)	0.056 (4)	0.045 (3)

### Geometric parameters (Å, º)

**Table:**

Cu1—O1	1.9530 (16)	O4—C8	1.243 (3)
Cu1—O2^i^	1.9535 (16)	O4—Cu1^i^	1.9572 (17)
Cu1—O4^i^	1.9572 (17)	C8—C9	1.493 (3)
Cu1—O3	1.9590 (16)	C9—C14	1.381 (3)
Cu1—O5	2.1334 (15)	C9—C10	1.387 (3)
Cu1—Cu1^i^	2.5905 (4)	C10—C11	1.387 (3)
O1—C1	1.245 (3)	C10—H10	0.9300
O2—C1	1.246 (3)	C11—C12	1.363 (4)
O2—Cu1^i^	1.9535 (16)	C11—H11	0.9300
C1—C2	1.493 (3)	C12—C13	1.373 (4)
C2—C3	1.380 (3)	C12—Cl2	1.740 (3)
C2—C7	1.394 (3)	C13—C14	1.380 (4)
C3—C4	1.384 (3)	C13—H13	0.9300
C3—H3	0.9300	C14—H14	0.9300
C4—C5	1.379 (4)	O5—C15	1.408 (4)
C4—H4	0.9300	O5—H5	0.8200
C5—C6	1.368 (4)	C15—C16	1.398 (6)
C5—Cl1	1.738 (2)	C15—H15A	0.9700
C6—C7	1.388 (3)	C15—H15B	0.9700
C6—H6	0.9300	C16—H16A	0.9600
C7—H7	0.9300	C16—H16B	0.9600
O3—C8	1.248 (3)	C16—H16C	0.9600
			
O1—Cu1—O2^i^	168.55 (7)	C8—O3—Cu1	123.45 (14)
O1—Cu1—O4^i^	91.24 (10)	C8—O4—Cu1^i^	122.94 (15)
O2^i^—Cu1—O4^i^	89.13 (10)	O4—C8—O3	124.7 (2)
O1—Cu1—O3	88.85 (9)	O4—C8—C9	118.06 (19)
O2^i^—Cu1—O3	88.55 (9)	O3—C8—C9	117.20 (19)
O4^i^—Cu1—O3	168.69 (7)	C14—C9—C10	119.4 (2)
O1—Cu1—O5	94.39 (7)	C14—C9—C8	120.0 (2)
O2^i^—Cu1—O5	96.99 (7)	C10—C9—C8	120.6 (2)
O4^i^—Cu1—O5	94.10 (7)	C11—C10—C9	120.0 (2)
O3—Cu1—O5	97.17 (7)	C11—C10—H10	120.0
O1—Cu1—Cu1^i^	85.05 (5)	C9—C10—H10	120.0
O2^i^—Cu1—Cu1^i^	83.59 (5)	C12—C11—C10	119.2 (2)
O4^i^—Cu1—Cu1^i^	84.70 (5)	C12—C11—H11	120.4
O3—Cu1—Cu1^i^	84.04 (5)	C10—C11—H11	120.4
O5—Cu1—Cu1^i^	178.66 (5)	C11—C12—C13	121.8 (2)
C1—O1—Cu1	122.56 (15)	C11—C12—Cl2	119.2 (2)
C1—O2—Cu1^i^	124.19 (14)	C13—C12—Cl2	119.0 (2)
O1—C1—O2	124.5 (2)	C12—C13—C14	118.9 (3)
O1—C1—C2	117.91 (19)	C12—C13—H13	120.5
O2—C1—C2	117.55 (18)	C14—C13—H13	120.5
C3—C2—C7	119.7 (2)	C13—C14—C9	120.5 (3)
C3—C2—C1	120.6 (2)	C13—C14—H14	119.7
C7—C2—C1	119.6 (2)	C9—C14—H14	119.7
C2—C3—C4	120.6 (2)	C15—O5—Cu1	121.5 (2)
C2—C3—H3	119.7	C15—O5—H5	109.5
C4—C3—H3	119.7	Cu1—O5—H5	125.7
C5—C4—C3	118.8 (2)	C16—C15—O5	119.3 (4)
C5—C4—H4	120.6	C16—C15—H15A	107.5
C3—C4—H4	120.6	O5—C15—H15A	107.5
C6—C5—C4	121.7 (2)	C16—C15—H15B	107.5
C6—C5—Cl1	119.0 (2)	O5—C15—H15B	107.5
C4—C5—Cl1	119.3 (2)	H15A—C15—H15B	107.0
C5—C6—C7	119.5 (2)	C15—C16—H16A	109.5
C5—C6—H6	120.3	C15—C16—H16B	109.5
C7—C6—H6	120.3	H16A—C16—H16B	109.5
C6—C7—C2	119.7 (2)	C15—C16—H16C	109.5
C6—C7—H7	120.2	H16A—C16—H16C	109.5
C2—C7—H7	120.2	H16B—C16—H16C	109.5
			
O2^i^—Cu1—O1—C1	−7.8 (6)	O5—Cu1—O3—C8	176.32 (19)
O4^i^—Cu1—O1—C1	83.9 (2)	Cu1^i^—Cu1—O3—C8	−3.11 (19)
O3—Cu1—O1—C1	−84.8 (2)	Cu1^i^—O4—C8—O3	−3.1 (4)
O5—Cu1—O1—C1	178.1 (2)	Cu1^i^—O4—C8—C9	176.85 (15)
Cu1^i^—Cu1—O1—C1	−0.6 (2)	Cu1—O3—C8—O4	4.6 (4)
Cu1—O1—C1—O2	−1.3 (4)	Cu1—O3—C8—C9	−175.33 (14)
Cu1—O1—C1—C2	179.60 (15)	O4—C8—C9—C14	−5.8 (3)
Cu1^i^—O2—C1—O1	3.4 (4)	O3—C8—C9—C14	174.2 (2)
Cu1^i^—O2—C1—C2	−177.55 (15)	O4—C8—C9—C10	175.3 (2)
O1—C1—C2—C3	−5.4 (3)	O3—C8—C9—C10	−4.7 (3)
O2—C1—C2—C3	175.4 (2)	C14—C9—C10—C11	−0.2 (4)
O1—C1—C2—C7	174.1 (2)	C8—C9—C10—C11	178.7 (2)
O2—C1—C2—C7	−5.0 (3)	C9—C10—C11—C12	−0.2 (4)
C7—C2—C3—C4	0.5 (4)	C10—C11—C12—C13	0.3 (5)
C1—C2—C3—C4	−180.0 (2)	C10—C11—C12—Cl2	−178.6 (2)
C2—C3—C4—C5	−0.2 (4)	C11—C12—C13—C14	0.0 (5)
C3—C4—C5—C6	−0.6 (4)	Cl2—C12—C13—C14	178.9 (3)
C3—C4—C5—Cl1	−179.7 (2)	C12—C13—C14—C9	−0.4 (5)
C4—C5—C6—C7	1.1 (4)	C10—C9—C14—C13	0.5 (4)
Cl1—C5—C6—C7	−179.76 (19)	C8—C9—C14—C13	−178.4 (3)
C5—C6—C7—C2	−0.8 (4)	O1—Cu1—O5—C15	−43.4 (3)
C3—C2—C7—C6	0.0 (4)	O2^i^—Cu1—O5—C15	137.8 (3)
C1—C2—C7—C6	−179.5 (2)	O4^i^—Cu1—O5—C15	48.1 (3)
O1—Cu1—O3—C8	82.0 (2)	O3—Cu1—O5—C15	−132.8 (3)
O2^i^—Cu1—O3—C8	−86.8 (2)	Cu1—O5—C15—C16	−179.0 (4)
O4^i^—Cu1—O3—C8	−8.6 (6)		

Symmetry code: (i) −*x*+2, −*y*, −*z*.

### Hydrogen-bond geometry (Å, º)

**Table:**

*D*—H···*A*	*D*—H	H···*A*	*D*···*A*	*D*—H···*A*
O5—H5···O2^ii^	0.82	2.35	3.073 (3)	148
O5—H5···O3^iii^	0.82	2.57	3.047 (3)	118

Symmetry codes: (ii) *x*−1, *y*, *z*; (iii) −*x*+1, −*y*, −*z*.
